# Blood viscosity during coagulation at different shear rates

**DOI:** 10.14814/phy2.12065

**Published:** 2014-07-03

**Authors:** Marco Ranucci, Tommaso Laddomada, Matteo Ranucci, Ekaterina Baryshnikova

**Affiliations:** 1 Department of Cardiothoracic, Vascular Anesthesia and Intensive Care, IRCCS Policlinico San Donato, Milan, Italy

**Keywords:** Blood viscosity, coagulation, gel point

## Abstract

During the coagulation process, blood changes from a liquid to a solid gel phase. These changes are reflected by changes in blood viscosity; however, blood viscosity at different shear rates (SR) has not been previously explored during the coagulation process. In this study, we investigated the viscosity changes of whole blood in 10 subjects with a normal coagulation profile, using a cone‐on‐plate viscosimeter. For each subject, three consecutive measurements were performed, at a SR of 20, 40, 80 sec^−1^. On the basis of the time‐dependent changes in blood viscosity, we identified the gel point (GP), the time‐to‐gel point (TGP), the maximum clot viscosity (MCV), and the clot lysis half‐time (CLH). The TGP significantly (*P* = 0.0023) shortened for increasing SR, and was significantly associated with the activated partial thromboplastin time at a SR of 20 sec^−1^ (*P* = 0.038) and 80 sec^−1^ (*P* = 0.019). The MCV was significantly lower at a SR of 80 sec^−1^ versus 40 sec^−1^ (*P* = 0.027) and the CLH significantly (*P* = 0.048) increased for increasing SR. These results demonstrate that measurement of blood viscosity during the coagulation process offers a number of potentially useful parameters. In particular, the association between the TGP and the activated partial thromboplastin time is an expression of the clotting time (intrinsic and common pathway), and its shortening for increasing SR may be interpreted the well‐known activating effects of SR on platelet activation and thrombin generation. Further studies focused on the TGP under conditions of hypo‐ or hypercoagulability are required to confirm its role in the clinical practice.

## Abstract (precis)

Measurement of blood viscosity with cone‐on‐plate technology allows to detect the transition point between liquid and gel phases of blood during coagulation (gel point). The time‐to‐gel point is dependent on the shear rate and demonstrated an association with the activated partial thromboplastin time.

## Introduction

Fluid viscosity is the ratio between shear stress and shear rate (SR). For Newtonian fluids, it linearly decreases for increased SRs. For non‐Newtonian fluids, like whole blood, the inverse relationship between viscosity and SR is maintained, following a non‐linear function, and the main determinants of blood viscosity are the hematocrit and the temperature.

Shear rates is an important factor within the coagulation process of blood. Platelet adhesion and activation are strongly dependent on SR (Chen et al. 2013; Sheriff et al. 2013; Kragh et al. 2014; Li et al. 2014), and thrombin generation is increased under conditions of high SR (Haynes et al. 2011).

Whole‐blood coagulation at different SRs has been addressed in different studies, using different technologies (Evans et al. 2008). During the coagulation process, blood changes its physical properties from the status of a pregel viscoelastic fluid to a viscoelastic solid. The turning point between the two physical conditions has been previously defined as the *gel point* (GP) (Blomback and Bark 2004; Evans et al. 2008, 2010). Before reaching the GP, blood reacts to changes in SR as a non‐Newtonian fluid, exerting its viscous properties; after the GP, the forming clot under flow conditions manifests both the properties of a fluid (viscosity) and of a solid (elasticity), and these mixed characteristics take the name of *viscoelastic* properties of the clot.

Platelet adhesion and activation and thrombin generation are the major determinants of the time to reach the GP, since it is nowadays established that the large amounts of thrombin required to activate fibrinogen to fibrin and the subsequent formation of a stable fibrin‐platelet clot, are mainly generated on the platelet surface. Additionally, the clot strength, defined by its viscoelastic properties, depends on the combined effects of fibrinogen and platelets.

At present, the existing point‐of‐care (POC) tests are based on a measure of the viscoelastic properties of the clot. Thromboelastography (TEG, Haemonetics Corporation, Braintree, MA) and thromboelastometry (ROTEM, Tem International GmbH, Munich) are the most commonly used; however, they provide a measure of the viscoelastic properties that is expressed in arbitrary units (mm), based on the transmission of the movement of blood to a pin. In these tests, the SR is fixed at a very low value (0.5 sec^−1^) that is not found in any part of the natural circulation. Other less commonly used devices like the ReoRox (MediRox AB, Nyköping, Sweden) provide a separate measure of the viscous and elastic properties of the clot, however, again at a fixed SR of 69 sec^−1^. Measurement of viscosity during whole‐blood coagulation at different SRs has not been applied in the clinical setting.

We hypothesized that, given the relationship between SR and platelet adhesion/activation, measuring blood viscosity during coagulation at different SRs may provide a measure of the time‐to‐gel point (TGP). We therefore designed an experiment based on the measurement of blood viscosity during coagulation at different SRs, with an estimate of a number of viscosity‐dependent parameters, to confirm our hypothesis.

## Methods

### Study design and ethics

The study was conducted at the University Hospital IRCCS Policlinico San Donato and was supported by local research funds. The study design was approved by the Ethics Committee of the San Raffaele Hospital, and all the patients signed a written informed consent.

### Subject population

Ten patients undergoing minor vascular surgery (saphenectomy), not pretreated with anticoagulants or antiplatelet agents, and with a normal coagulation profile at the preoperative laboratory assays.

### Blood samples and routine coagulation tests

The day before surgery, the subjects received the standard set of blood laboratory assays, which includes a routine screening of coagulation tests: activated partial thromboplastin time (aPTT); international normalized ratio (INR) for prothrombin time; platelet count (cells·*μ*L^−1^). Hematocrit (%) and hemoglobin values (g·dL^−1^) were routinely collected.

### Viscosity tests

For all the viscosity tests, 2 mL of whole blood was collected from the antecubital vein on the day of surgery, inside the operating theater. Immediately after withdrawal, 1 mL of whole blood was placed into kaolin primed tubes (Haemonetics Corporation, Braintree, MA) and tested for viscosity using a cone‐on‐plate Brookfield DV3T viscosimeter (Brookfield Engineering Laboratories, Stoughton, MA). Cone‐on‐plate viscosimetry is based on a system that uses a cone of very shallow angle in contact with a flat plate. The cone rotates on the plate where an amount of fluid is placed. By changing the size of the cone and the rotational speed different shear rates may be applied to the fluid. The shear rate beneath the plate is constant; a graph of shear stress (torque) against shear rate (angular velocity) yields the viscosity. A quantity of 500 *μ*L of blood was placed on the surface of the plate using a calibrated micropipette; the surface of the cone‐on‐plate system was maintained at a temperature of 37°C using a heat‐exchanger Heater Unit HU 35 Maquet (Getinge Group, Getinge, Sweden). The spindle of the cone‐on‐plate system was equipped with a cone model CPA‐52Z.

Three sequential viscosity tests were performed, each one on newly withdrawn blood from the same subject. Test 1 was performed at a rotating speed of the cone on the plate of 10 min^−1^, correspondent to a SR of 20 sec^−1^; test 2 at a rotating speed of 20 min^−1^, correspondent to a SR of 40 sec^−1^; and test 3 at a rotating speed of 40 min^−1^, correspondent to a SR of 80 sec^−1^. During each test, and at an interval of 10 sec, the values of shear stress were recorded and the correspondent viscosity value (centipoise, cP) calculated with a specific software on a computer. Each tests lasted from a minimum of 10 min (60 measurements) to a maximum of 30 min (180 measurements), depending on the time required to calculate all the derived variables.

### Calculations, parameters definition, and statistical analysis

**Figure 1.**
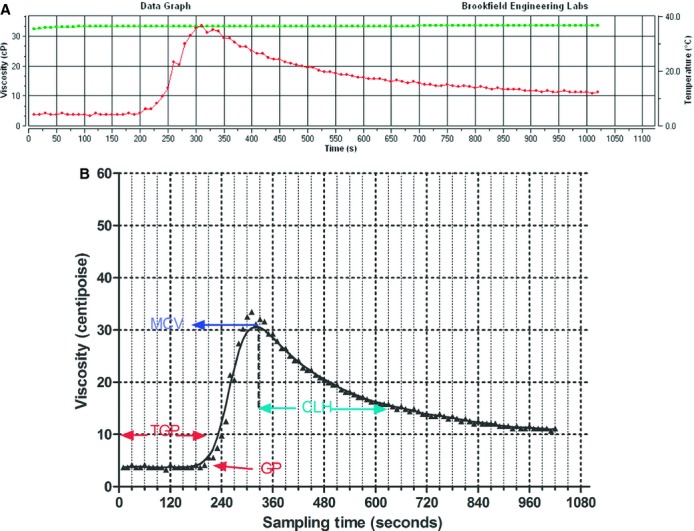
(A) output of a viscosity time course during the experimental procedure; green line, temperature; red line, viscosity; cP, centipoise. (B) locally weighted scatterplot smoothing of the data reported in part A, with viscosity‐derived parameters; CLH, clot lysis half‐time; GP, gel point; MCV, maximum clot viscosity; TGP, time‐to‐gel point.

**Figure 2.**
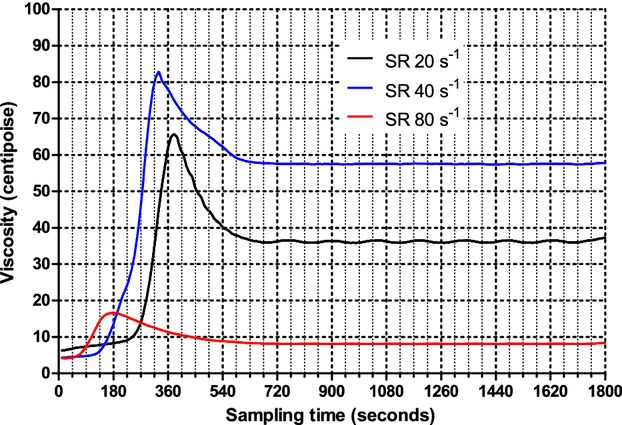
Viscosity time course during the experimental procedure in the same subject at different shear rates. SR, shear rate.

The output of the measurements was produced in tabular view using an Excel file (Microsoft, Redmond, WA) and simultaneously in graphical form (Fig. 1A). After completion of the test, data from the Excel file were merged into a computerized statistical package Prism 6.0 (GraphPad, San Diego, CA). Using a locally weighted scatterplot smoothing technique, a function approximating data from the test was derived, providing a graphical analysis of the blood viscosity changes over time, during the coagulation process (Fig. 1B). An example of the three tests in one of the subjects is reported in Fig. 2.

On the basis of the previously described analysis, the following parameters were defined:

1. The value of blood viscosity measured during the liquid phase of blood coagulation (before the GP) was defined as baseline blood viscosity (BV).
2. The GP is the transition point between the liquid and gel phase of the blood. It was defined as the last viscosity value measured before the first increase in viscosity of at least 20% more than the previous point, followed by a progressive viscosity increase for the next points in time.
3. The TGP is defined as the time (sec) from test initiation to the GP.
4. The maximum clot viscosity (MCV) was defined as the peak point of clot viscosity.
5. The clot lysis halftime (CLH) was defined as the time required to decrease clot viscosity from the peak value to a value 50% lower than the peak value. When this value was not reached after 30 min, it was arbitrarily settled at 30 min minus the time at which the MCV was reached.

The value of the above parameters for each patient and each test at different SRs was entered into a computerized statistical package (SPSS 20.0, IBM, Chicago, IL) together with the routine coagulation tests.

Data are expressed as mean and standard deviation or as mean and standard error. Differences within subjects and between values obtained at different SRs were tested with an analysis of variance for repeated measurements with post hoc Tukey's test. Association between routine coagulation tests and the viscosity‐related parameters were explored using regression analyses. Different equations were tested (linear, logarithmic, quadratic, and cubic) and the function with the best fit was chosen to define the phenomenon. A *P* value < 0.05 was considered statistically significant.

## Results

Values of routine coagulation tests in the subject population are shown in Table 1. With the only exception of one subject with an aPTT slightly shorter (24.7 sec) than the lower limit of the normal range, all the variables were within the normal range, thus confirming that the subject population was representative of a normal coagulation profile. Platelet function testing was no part of standard coagulation testing.

**Table 1.** Details of routine coagulation tests and hematocrit values in the subject population (*N* = 10).

Variable	Mean (SD)	Range	Normal range
Platelet count (×1000 cells·*μ*L^−1^)	216 (65)	159–354	150–400
INR	1.03 (0.08)	0.95–1.18	0.8–1.1
aPTT (sec)	28.4 (2.6)	24.7–32.8	25–35
Hematocrit (%)	39.2 (3.5)	38.0–45.4	38.8–50 (male) 34.5–44.5 (female)

aPTT, activated partial thromboplastin time; INR, international normalized ratio; SD, standard deviation.

**Figure 3.**
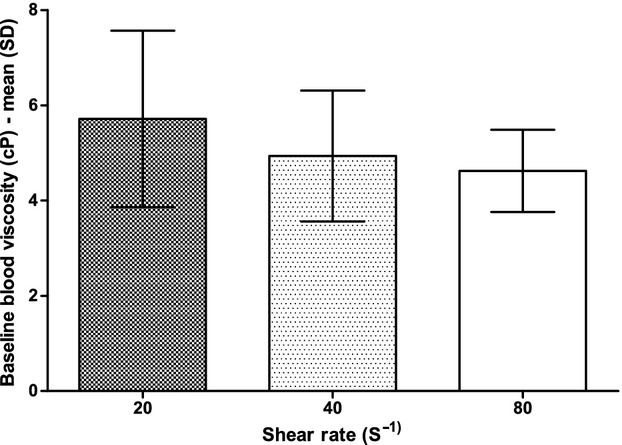
Baseline blood viscosity at different shear rates. cP, centipoise; SD, standard deviation

**Figure 4.**
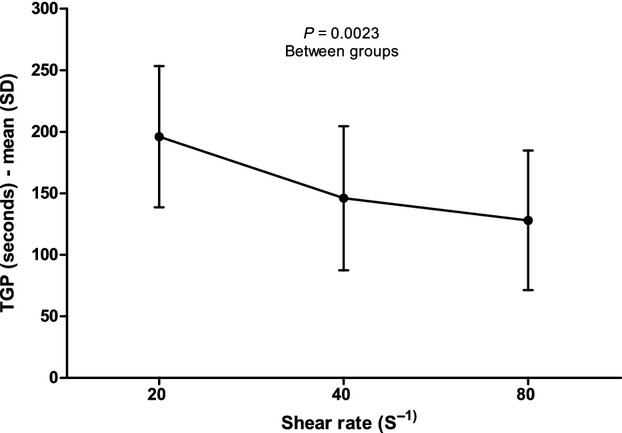
Time‐to‐gel point at different shear rates. SD, standard deviation; TGP, time‐to‐gel point.

The baseline BV, measured when the whole blood was in liquid phase, did not change under different conditions of SR (Fig. 3). TGP decreased for increasing values of SR (Fig. 4) with a significant (*P* = 0.0023) difference between groups; at the post hoc test, TGP at a SR of 80 sec^−1^ (128 ± 57 sec) was shorter than at a SR of 20 sec^−1^ (196 ± 57 sec, *P* < 0.01) and TGP at a SR of 40 sec^−1^ (146 ± 59 sec) was shorter than TGP at a SR of 20 sec^−1^ (*P* < 0.05).

**Figure 5.**
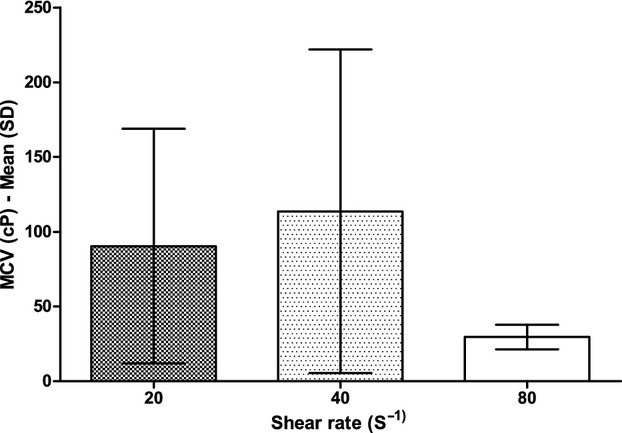
Maximum clot viscosity at different shear rates. cP, centipoise; MCV, maximum clot viscosity; SD, standard deviation.

Maximum clot viscosity (Fig. 5) was not different between SRs of 20 sec^−1^ and 40 sec^−1^, whereas it was significantly lower at SR of 80 sec^−1^ (30 ± 8 cP) versus a SR of 40 sec^−1^ (114 ± 108 cP, *P* = 0.027).

**Figure 6.**
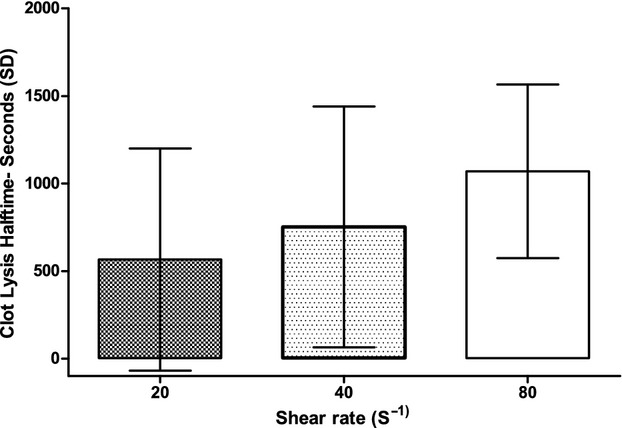
Clot lysis half‐time at different shear rates. SD, standard deviation.

Clot lysis half‐time (Fig. 6) significantly (*P* = 0.048) increased for increasing values of SR, with a difference for a SR of 80 sec^−1^ (1069 ± 495 sec) versus 20 sec^−1^ (566 ± 634 sec, *P* < 0.05).

**Figure 7.**
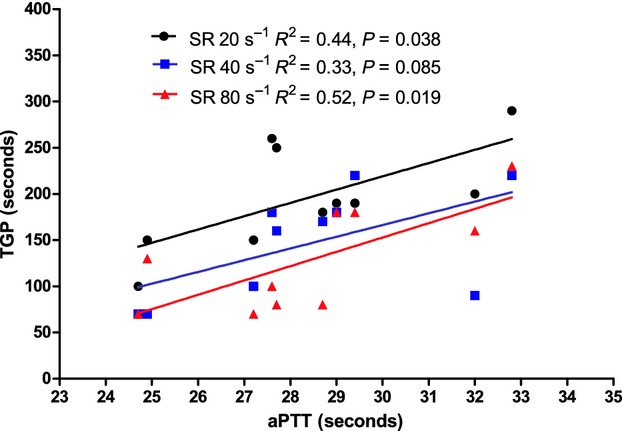
Linear regression relationship between activated partial thromboplastin time and time‐to‐gel point at different shear rates. aPTT, activated partial thromboplastin time; SR, shear rate; TGP, time‐to‐gel point.

No significant association was found among INR, platelet count, and hematocrit and any of the viscosity‐dependent parameters measured at any level of SR. Conversely (Fig. 7), the TGP was significantly associated with the aPTT at SRs of 20 sec^−1^ and 80 sec^−1^, with a trend toward association at a SR of 40 sec^−1^. By pooling all the experimental points together, there is a significant (*P* = 0.021) linear association between SR and TGP, defined by the equation: TGP (sec) = 205 – 1.036. SR (sec^−1^).

## Discussion

Our study demonstrates that the measurement of whole‐blood viscosity allows the determination of the GP during the coagulation process, being sensitive to the SR‐dependent changes in the TGP. Additionally, even in a range of normal aPTT, the TGP determined by blood viscosity measurement reflects differences in aPTT, being therefore associated with different degrees of the intrinsic and common pathway activation.

After reaching the GP, the measurement of the viscous component of the forming clot offers an additional information in terms of clot lysis dynamics, with a prolonged CLH for increasing SRs, and with a lower degree of viscosity when a SR of 80 sec^−1^ is applied.

However, the interpretation of the phenomenon before (TGP) and after (MCV, CLH) the gel point reflects different aspects of the coagulation process. TGP reflects thrombin generation, which in turn is strongly dependent on platelet activation. SR is a powerful platelet activator; therefore, we could identify a linear relationship between SR and TGP.

Maximum clot viscosity is a measure of clot firmness, which depends on platelet count, fibrinogen concentration, and factor XIII. CLH is a measure of clot stability, and again other factors (like plasmin generation) play a role. Therefore, our experimental setting was probably inadequate to elucidate the clinical value of these parameters.

The time required to convert fibrinogen into an efficient network of fibrin and platelets (generally defined as “clotting time”) is an important parameter in medicine, being associated with the occurrence of thromboembolic events (if shortened), or bleeding (if prolonged). Factors associated with a shortened coagulation time are congenital prothrombotic diseases and increased activity of coagulation factors as observed in diabetes (Kim et al. 2014), elderly patients (Weingarz et al. 2013), and other conditions. Factors associated with a prolonged clotting time are congenital coagulation disorders, like hemophilia, acquired consumption of coagulation factors, like in trauma‐induced coagulopathy and cardiac surgery with cardiopulmonary bypass, and the use of specific drugs, like warfarin, direct thrombin inhibitors, heparin. The assessment of the GP and the measure of the TGP as a marker of the clotting time are therefore relevant clinical parameters. The detection of the GP has been defined as a marker of an *incipient* clot formation (Evans et al. 2008).

In clinical practice, a fast assessment of the TGP at the point of care is certainly useful in case of conditions acutely affecting the coagulation process, like major cardiovascular surgery, liver surgery, trauma, postpartum hemorrhage, and others.

The most frequently used POC devices for coagulation assessment are TEG and ROTEM. Both the devices have shown to be useful in guiding diagnosis and therapy in different scenarios characterized by acute bleeding (Coakley et al. 2006; Tripodi et al. 2009; Görlinger et al. 2013; Tanaka et al. 2013). In these devices, the transition of whole blood from a viscoelastic fluid to a viscoelastic solid is reflected by the reaction time (R‐time) in TEG and the clotting time (CT) in ROTEM. A prolongation of the R‐time or the CT suggests a poor activity of soluble coagulation factors, due to consumption or to the effects of specific drugs (Ranucci et al. 2012).

However, and despite its clinical usefulness, TEG and ROTEM have some potentially important drawbacks. The strain amplitude experienced by the sample is not a constant and may change during the coagulation process, with an applied strain that decreases progressively as the system clots (Evans et al. 2008). In a recent study, the TGP measured with a rheometer was found associated with the aPTT in healthy subjects, whereas the R‐time at TEG was not (Evans et al. 2010). In surgical patients, the R‐time has only a 19% sensitivity to detect an aPTT longer than 40 sec (Ågren et al. 2013). Conflicting data exist as to the association between aPTT and the CT at ROTEM, with authors claiming an acceptable association (Theusinger et al. 2013), and others demonstrating no association (Haas et al. 2012).

In the present scenario, the assessment of the GP and the measure of the TGP with a direct cone‐on‐plate viscosimetry may be of relevant clinical importance, given the association between the TGP and the aPTT found at any level of SR, within a normal range of aPTT. At a SR of 80 sec^−1^, the GP was reached at a median value of 115 sec (range 70–230 sec), therefore offering a POC evaluation of the clotting time in <2 min. With respect to the other POC tests, it is of notice that cone‐on‐plate viscosimetry has a number of potential advantages, including (i) the application of a constant level of shear rate throughout the whole coagulation process, (ii) the measurement of viscosity using standard units (centipoise) rather than arbitrary units, (iii) the possibility to work at different SRs, and (iv) the application of a SR more adequately reflecting the physiological conditions of the circulation.

The values of BV obtained before the GP are higher than what usually reported for whole blood at a normal hematocrit and at 37°C of temperature. This is due to the effects of the low SR applied in our experimental procedure. At high SR in the range of 100–200 sec^−1^ (found at the arterial side of the circulation) whole‐blood viscosity is actually 4–5 cP; however, at lower SRs (in the range of 20–80 sec^−1^), it is reported as about 6–8 cP, consistent with our results (Baskurt and Meiselman 2003).

In the past, other devices exploring the rheological properties of blood during coagulation at an arterial SR have been proposed (Görög and Ahmed 1984). However, the complex technical requirements did not allow these instruments to enter the clinical practice.

To our knowledge, there is only one previous study using viscosity measurement (ultrasound‐based technique) during blood coagulation (Yesner et al. 1951), where viscosity changes over time reflected a behavior similar to what observed in our experiment. Interestingly, the authors found that viscosity was constant until a point where it increased, reaching a plateau. Subsequently, the plateau was maintained in some cases, whereas in others there was a time‐dependent decrease. The time to reach the point at which viscosity started raising was about 3 min in healthy subjects (equivalent to the measure of the TGP in our study), but this time was prolonged up to 15 min in a pseudohemophilic patient, whereas it came back to normal by infusing fresh frozen plasma. The viscosity at plateau in healthy patients was about 70 cP, again consistent with our MCV values. This value was decreased under conditions of hypocalcemia and bleeding. These results are confirmative that the measurement of whole‐blood viscosity during the coagulation process may offer parameters useful in daily clinical practice.

### Study limitations and future perspectives

There are some limitations in this study. Being a study dealing with a new technology, no adequate sample size calculation was possible; additionally, the blood samples were not randomized for the different SRs, and the number of SRs tested was limited to three. This limited the analysis to SRs that in the natural circulation are reached at a venous, but not at an arterial level. The definition of the GP in our study was arbitrarily settled at the point when the whole blood, subjected to a constant SR, starts increasing its viscosity. We acknowledge that an exact measure of the GP in physical terms requires more sophisticated technologies. Fourier transform mechanical spectroscopy (Evans et al. 2010) and other complex methods have been proposed. However, the application of these technologies is difficult to hypothesize in the clinical environment and moreover in the setting of POC tests. Additionally, the use of viscosity‐based techniques to approximate the GP of polymers undergoing transition from the liquid to the solid state at different temperatures has been proposed in rheological studies in the past (Apicella et al. 1984).

The results of this study in terms of GP determination with direct viscosimetry need to be confirmed under conditions that modify the clotting time. In particular, the adequacy of the TGP as a marker of whole‐blood clotting time should be tested under conditions of prolonged clotting time, as happens under the well‐known effects of unfractionated heparin, low‐molecular‐weight heparin, direct thrombin inhibitors, and warfarin. If confirmed within this clinical scenario, the assessment of the TGP with cone‐on‐plate viscosimetry could enter into the armamentarium of coagulation POC tests, given its rapid response and feasibility at the bedside. Further studies in this direction are warranted.

## Conflict of Interest

None declared.
